# Discrimination between hypervirulent and non-hypervirulent ribotypes of *Clostridioides difficile* by MALDI-TOF mass spectrometry and machine learning

**DOI:** 10.1007/s10096-023-04665-y

**Published:** 2023-09-18

**Authors:** Ahmed Mohamed Mostafa Abdrabou, Issa Sy, Markus Bischoff, Manuel J. Arroyo, Sören L. Becker, Alexander Mellmann, Lutz von Müller, Barbara Gärtner, Fabian K. Berger

**Affiliations:** 1 Institute of Medical Microbiology and Hygiene, Saarland University, Kirrberger Straße 100, Building 43, D-66421 Homburg, Saar Germany; 2 Medical Microbiology and Immunology Department, Faculty of Medicine, Mansoura University, El Gomhouria Street, Mansoura, 35516 Egypt; 3 National Reference Center for Clostridioides (Clostridium) difficile, Homburg-Münster-Coesfeld, Germany; 4 Clover Bioanalytical Software, Av. del Conocimiento, 41, 18016 Granada, Spain; 5 Institute of Hygiene, University of Münster, Robert-Koch-Straße 41, 48149 Münster, Germany; 6 Christophorus Kliniken Coesfeld, Coesfeld, Germany

**Keywords:** *Clostridium difficile*, Ribotypes, Anaerobic bacteria, MALDI-TOF mass spectrometry, Proteomic signature, Machine learning, Identification

## Abstract

Hypervirulent ribotypes (HVRTs) of *Clostridioides difficile* such as ribotype (RT) 027 are epidemiologically important. This study evaluated whether MALDI-TOF can distinguish between strains of HVRTs and non-HVRTs commonly found in Europe. Obtained spectra of clinical *C. difficile* isolates (training set, 157 isolates) covering epidemiologically relevant HVRTs and non-HVRTs found in Europe were used as an input for different machine learning (ML) models. Another 83 isolates were used as a validation set. Direct comparison of MALDI-TOF spectra obtained from HVRTs and non-HVRTs did not allow to discriminate between these two groups, while using these spectra with certain ML models could differentiate HVRTs from non-HVRTs with an accuracy >95% and allowed for a sub-clustering of three HVRT subgroups (RT027/RT176, RT023, RT045/078/126/127). MALDI-TOF combined with ML represents a reliable tool for rapid identification of major European HVRTs.

**Supplementary Information:**

The online version contains supplementary material available at 10.1007/s10096-023-04665-y.

## Introduction

*Clostridioides difficile* is a significant cause of nosocomial diarrhea in industrialized nations [1]. Hypervirulent ribotypes (HVRTs) such as RT027 have influenced the global molecular epidemiology of *C. difficile* [2] leading to a higher disease burden [3]. RT027 has caused numerous outbreaks in Europe and the USA [4]. However, on a global scale, other HVRTs exist, e.g., RT023 being considered an emerging HVRT [5], and RT045 that might confer a zoonotic potential [6]. Besides the toxins A and B (genes: *tcdA*, *tcdB*) destroying the actin cytoskeleton, HVRT strains usually harbor a third toxin (binary toxin, gene: *cdtAB*) that increases bacterial adhesion through microtubular protrusions [7, 8].

Several typing techniques have been developed to identify RTs of higher importance. These include in particular ribotyping [9] and whole genome sequencing (WGS) [10]. However, both methods are comparably time- and resource-consuming and therefore usually not available in most laboratories. Matrix-assisted laser desorption ionization time-of-flight (MALDI-TOF) mass spectrometry (MS) is widely distributed and an easy-to-use tool for the identification of bacteria [11], which is also used for bacterial subtyping [12].

Machine learning (ML) can further expand its capabilities, by training algorithms on a variety of databases garnered from analysis of bacterial proteins. The process can become increasingly automated and more accurate in identifying bacteria [13]. MALDI-TOF can distinguish several important RTs, such as RT001 [14, 15], RT017 [16], RT027/RT176 [14, 15, 17], and RT078/RT126 [15].

This study aimed to establish and evaluate a combined MS/ML protocol to rapidly distinguish between major HVRTs and non-HVRTs of high epidemiologic importance in Europe.

## Material and methods

### Strain collection and cultivation

Two hundred forty clinical *C. difficile* isolates (157 training set and 83 validation set) from the German National Reference Center’s strain collection were tested (Table 1) [18]. Strains were pre-characterized by PCR-ribotyping with their selection based on their epidemiologic importance in Europe (Supplementary File S1).

**Table 1:** Number of strains included in this study. *HVR*, hypervirulent *C. difficile* strains; *non-HVR*, non-hypervirulent *C. difficile* strains

Group	Training set	Validation set	Total
HVR	65	39	104
Non-HVR	92	44	136
Total	157	83	240

For analysis, cryopreserved clinical isolates were thawed, sub-cultured on trypticase soy agar plates with 5% sheep blood (BD Biosciences, USA), and incubated at 37 °C for 48 h using an anaerobic chamber (Whitley, UK). Prior to further processing, fresh colonies underwent MALDI-TOF analysis for purity check (Bruker Daltonics, USA).

### Protein extraction, spectra acquisition, and species confirmation

Off-plate ethanol/formic acid protein extraction protocol was used as described previously [19]. Briefly, 2–3 colonies were suspended in 300-μL liquid chromatography (LC-MS) grade water (Merck, Germany). Next, 900-μL absolute ethanol (Merck) were added followed by vortexing, then centrifuged (18,000 × g for 2 min). The supernatant was discarded and the bacterial pellet was completely dried. Cells were resuspended in 10 μL of 70% (v/v) formic acid and 10 μL of acetonitrile and thoroughly mixed and centrifuged (see above). One μL of the cleared supernatant was spotted four times (technical replicates) on the target plate. After air-drying, each spot was covered with 1 μL of saturated α-cyano-4-hydroxy-cinnamic acid (HCCA) matrix solution (Bruker). Measurements were performed with the Microflex LT smart mass spectrometer using the AutoXecute algorithm implemented in the Flexcontrol software (v.3.4, Bruker). To ensure biological reproducibility, this procedure was repeated with a new subculture of each isolate. Bacterial test standard (BTS, Bruker) was used for calibration. For species confirmation, acquired spectra were compared to the Bruker BDAL database (10,184 species-specific main spectra profiles) using the MALDI Biotyper compass explorer software (v.3.0).

### MALDI-TOF parameters

Two hundred forty laser shots (40 shots each at 6 random positions) were used to generate spectra profiles in linear positive ion mode (laser frequency 200 Hz), high voltage (20 kV), and pulsed ion extraction (520 ns). The mass-to-charge ratio (*m/z*) ranged between 2 and 20 kDa.

### Spectra analysis

Raw spectra were visualized using the FlexAnalysis software (Bruker), then exported to the Clover MS Data Analysis Software [20].

All spectra were preprocessed using default parameters: Smoothing (Savitzky–Golay filter: window length 11, polynomial order: 3); baseline removal (method: top-hat filter, factor 0.02); replicates alignment (constant tolerance: 0.2, linear tolerance: 2000 ppm) [21]. Obtained spectra from technical and biological replicates were combined to create one average spectrum per isolate that were used as input for generating peak matrices.

### Classification using machine learning algorithms

The Clover Biosoft platform was used for ML analyses utilizing pre-processed spectra. Firstly, spectra of 157 training set samples (Table 1) were used to distinguish between HVRTs and non-HVRTs. Three peak matrices were generated using different methods as previously described [21]. The “full spectrum method” uses each mass every 0.5 Da, regardless of its intensity, followed by a total ion current (TIC) normalization of the peak intensities. The “threshold method” (factor 0.01) excluded all peaks with an intensity <1% of the maximum intensity seen in each spectral profile and was coupled with a TIC normalization either before (TICp) or after (pTIC) removal of the minor peaks. For the individual peak identification in spectral profiles, a constant tolerance of 0.5 Da and linear tolerance of 500 ppm was applied [21]. All generated peak matrices were used as input for ML analyses utilizing unsupervised and supervised algorithms [22]. As an unsupervised algorithm, principal component analysis (PCA) was tested. For supervised algorithms, support vector machine (SVM), partial least square discriminant analysis (PLS-DA), k-nearest neighbor (KNN), and random forest (RF) were utilized. For internal validation, a 10-fold cross-validation was applied. Based on cross-validation results, confusion matrix, area under receive operating characteristic (AUROC) curve, and area under precision recall (AUPR) curve were used to estimate the prediction models’ performance. Secondly, HVRTs pre-processed spectra only were used for MS/ML subtyping.

### External validation

The two best performing models in the cross validation (Table 2) were externally validated using pre-processed spectra of 83 new clinical isolates (validation set, Table 1) to evaluate their reliability and robustness.

**Table 2:** Confusion matrix of 10-fold cross-validation results: classification scores (in %) obtained with four different supervised ML algorithms (RF, PLS-DA, KNN, and SVM). *HVR*, hypervirulent; *non-HVR*, non-hypervirulent; *RT*, ribotypes. HVR RTs group is the selected category (positive category); *TP*, true positive; *FP*, false positive; *PPV*, positive predictive value; *TN*, true negative; *FN*, false negative; *NPV*, negative predictive value

Actual/predicted	HVR RTs	Non-HVR RTs	% Correct
Support vector machine (SVM)			
HVR RTs	39 (TP)	26 (FN)	60.0% (sensitivity)
Non-HVR RTs	8 (FP)	84 (TN)	91.3% (specificity)
	83.0% (PPV)	76.4% (NPV)	78.3% (accuracy)
K-nearest neighbor (KNN)			
HVR RTs	58 (TP)	7 (FN)	89.2% (sensitivity)
Non-HVR RTs	4 (FP)	88 (TN)	95.7% (specificity)
	93.6% (PPV)	92.6% (NPV)	93.0% (accuracy)
Partial least square discriminant analysis (PLS-DA)			
HVR RTs	64 (TP)	1 (FN)	98.5% (sensitivity)
Non-HVR RTs	1 (FP)	91 (TN)	98.9% (specificity)
	98.5% (PPV)	98.9% (NPV)	98.7% (accuracy)
Random forest (RF)			
HVR RTs	64 (TP)	1 (FN)	98.5% (sensitivity)
Non-HVR RTs	0 (FP)	92 (TN)	100% (specificity)
	100% (PPV)	98.9% (NPV)	99.4% (accuracy)

## Results

### MALDI-TOF spectra acquisition

**Fig. 1:**
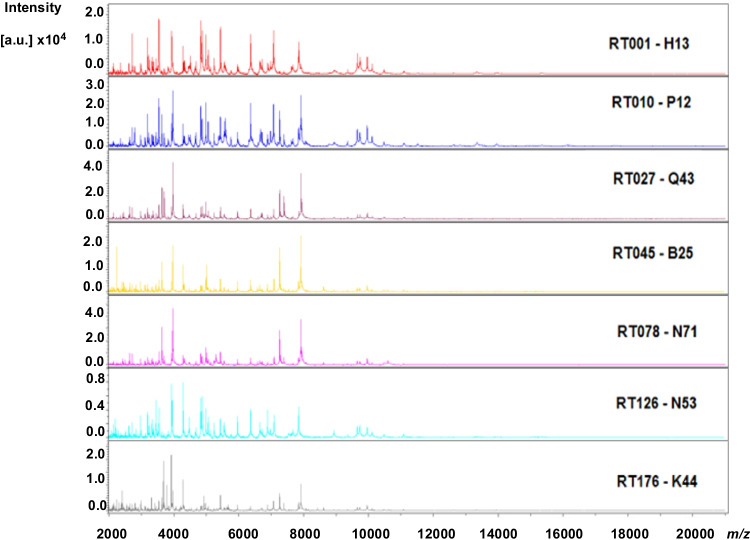
Representative spectral profiles of different ribotypes (RTs and corresponding internal code) of *C. difficile* utilized in this study. *X*-axis represents the mass-to-charge ratio (*m/z*), and *Y*-axis represents intensity values in arbitrary unit (a.u). RT, ribotype

Representative spectral profiles from different RTs are visualized in Fig. 1. Spectra of all isolates were correctly identified as *C. difficile* (Supplementary File S2).

### Discrimination between HVRTs and non-HVRTs

**Fig. 2:**
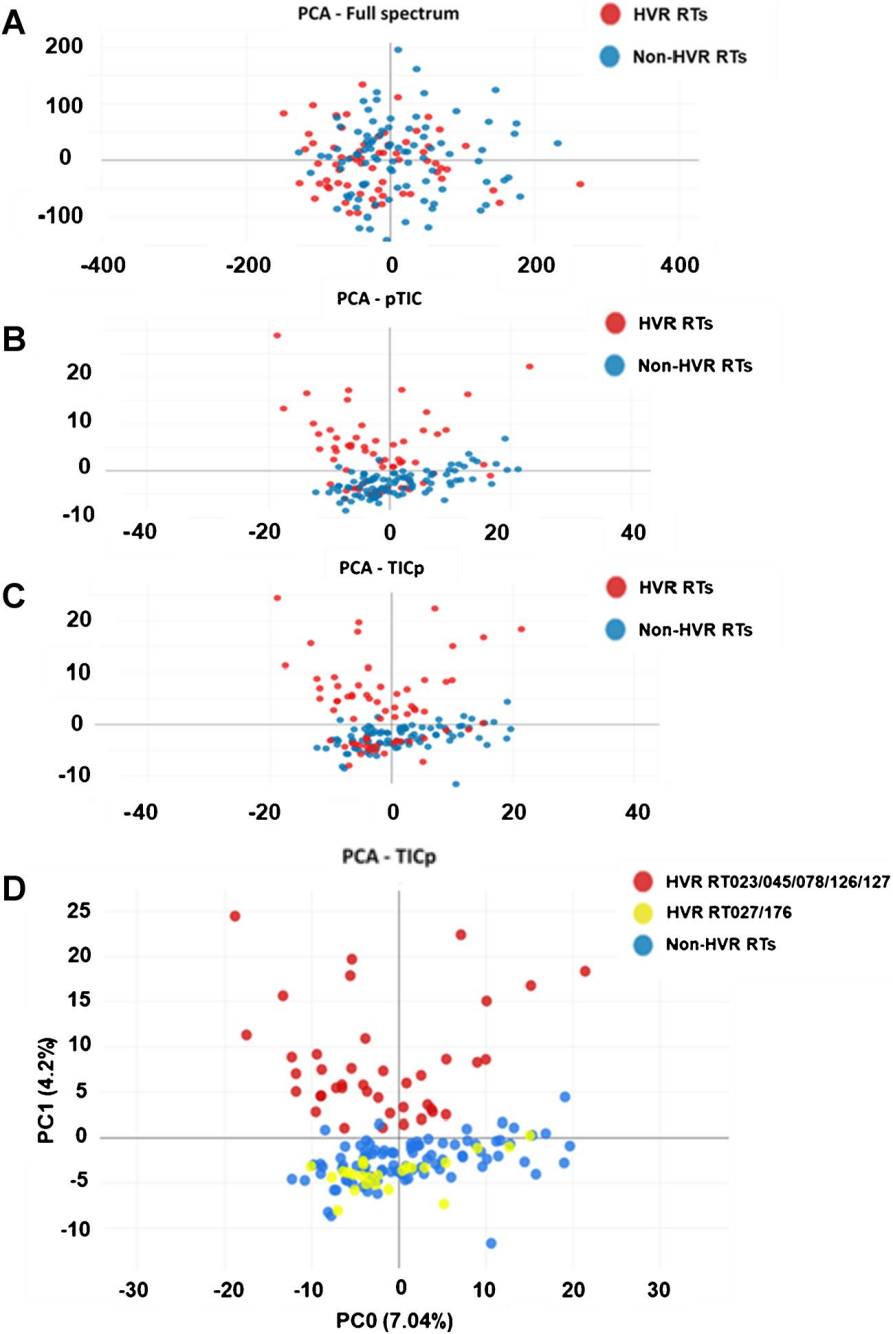
Classification applying an unsupervised algorithm: two-dimensional view of a principal component analysis (PCA) using “full spectrum” method (**A**), “threshold method” with normalization performed after peak finding (pTIC) (**B**), and “threshold method” with normalization performed before peak finding (TICp) (**C**). Each circle represented an individual *C. difficile* strain visualized with different colors associated with the RT group. HVR RTs, hypervirulent *C. difficile* ribotypes (RT023, RT027, RT045, RT078, RT126, RT127, and RT176) depicted in red; non-HVR RTs, non-hypervirulent ribotypes depicted in blue. For **D**, HVR strains were divided into two groups (RT023, RT045, RT078, RT126, and RT127 in red, while RT027 and RT176 are in yellow)

Average spectra of 157 isolates (training set) were used to create three different peak matrices being tested by PCA (Fig. 2). When using the “full spectrum method” for peak matrix generation, PCA failed to separate HVRT from non-HVRT isolates (Fig. 2A).

Better separation was achieved, when either of the two “threshold methods” (pTIC and TICp) was applied combined with PCA (Fig. 2B, C). However, these test procedures were still insufficient to reliably separate HVRTs from non-HVRTs due to a subset of HVRTs belonging to RT027/176 merging with non-HVRTs (Fig. 2).

**Fig. 3:**
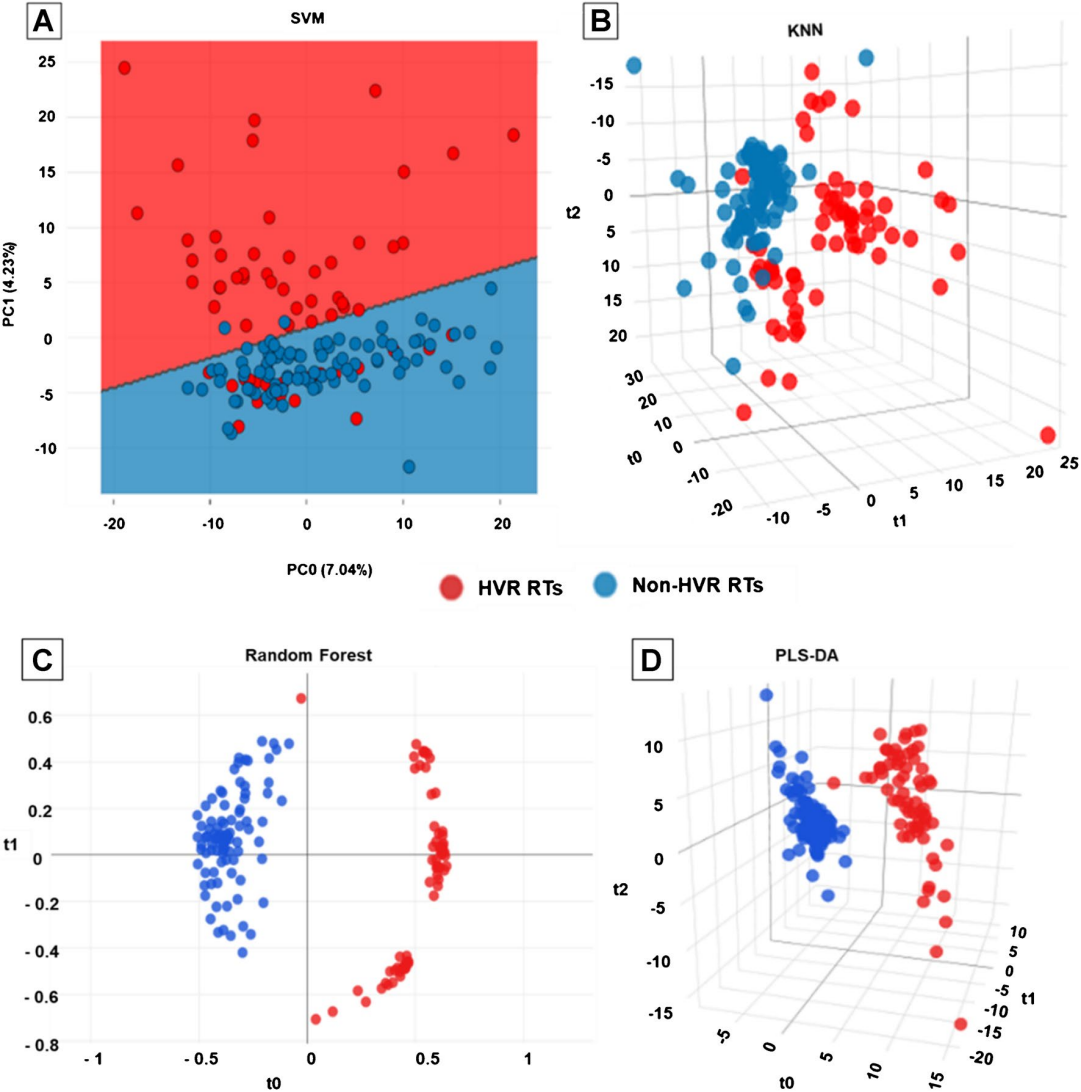
Classification of *C. difficile* strain using four supervised machine learning (ML) algorithms: support vector machine (SVM) (**A**), K-nearest neighbor (KNN) (**B**), random forest (RF) (**C**), and partial least square discriminant analysis (PLS-DA) (**D**). Each circle represented an individual *C. difficile* strain depicted with different colors associated with the RT group. HVR RTs, hypervirulent *C. difficile* ribotypes (RT023, RT027, RT045, RT078, RT126, RT127, and RT176) depicted in red; non-HVR RTs, non-hypervirulent ribotypes depicted in blue

The TICp method showed the best separation between both groups and was thus used for downstream supervised ML analyses. SVM classification results displayed again only partial discrimination between HVRT and non-HVRT strains, as RT027/176 isolates clustered mostly together with non-HVRTs (Fig. 3A). In contrast, RF, PLS-DA, and KNN prediction models allowed for a much better discrimination (Fig. 3B–D).

After 10-fold cross validation of the supervised ML models, an overall accuracy of 99.4% was observed for the RF model, 98.7% for the PLS-DA model, 93.0% for the KNN model, and 78.3% for the SVM model (Table 2). The superior performances of the RF and PLS-DA models to reliably discriminate between HVRTs and non-HVRTs were confirmed by the ROC and PR curves with respective mean values of AUROC and AUPRC of 0.98 and 0.99 for RF, 0.99 and 1 for PLS-DA, 0.94 and 0.96 for KNN, and 0.74 and 0.79 for SVM (Supplementary File S3).

### External validation

The two most discriminative algorithms (RF and PLS-DA) were next used for models’ external validation. When tested with the MALDI-TOF spectra of 83 new clinical *C. difficile* isolates (validation set) that were added blinded to the models. Both prediction models produced promising classification results with total accuracies of 98.8% (RF) and 97.6% (PLS-DA) (Table 3).

**Table 3:** External validation: classification scores (in %) of 83 new *C. difficile* strains by the two best supervised ML algorithms (RF and PLS-DA). *HVR*, hypervirulent; *non-HVR*, non-hypervirulent; *RTs*, ribotypes. HVR RTs group is the selected category (positive category); *TP*, true positive; *FP*, false positive; *PPV*, positive predictive value; *TN*, true negative; *FN*, false negative; *NPV*, negative predictive value

Actual/predicted	HVR RTs	Non-HVR RTs	% Correct
Partial least square discriminant analysis (PLS-DA)			
HVR RTs	38 (TP)	1 (FN)	97.4% (sensitivity)
Non-HVR RTs	1 (FP)	43 (TN)	97.7% (specificity)
	97.4% (PPV)	97.7% (NPV)	97.6% (accuracy)
Random forest (RF)			
HVR RTs	39 (TP)	0 (FN)	100% (sensitivity)
Non-HVR RTs	1 (FP)	43 (TN)	97.7% (specificity)
	97.5% (PPV)	100% (NPV)	98.8% (accuracy)

The respective mean values for AUROC and AUPRC confirmed the high performance of both models, with 0.98 and 0.92 (RF), and 0.96 and 0.97 (PLS-DA) (Supplementary File S4).

### ML-subtyping of HVRTs

**Fig. 4:**
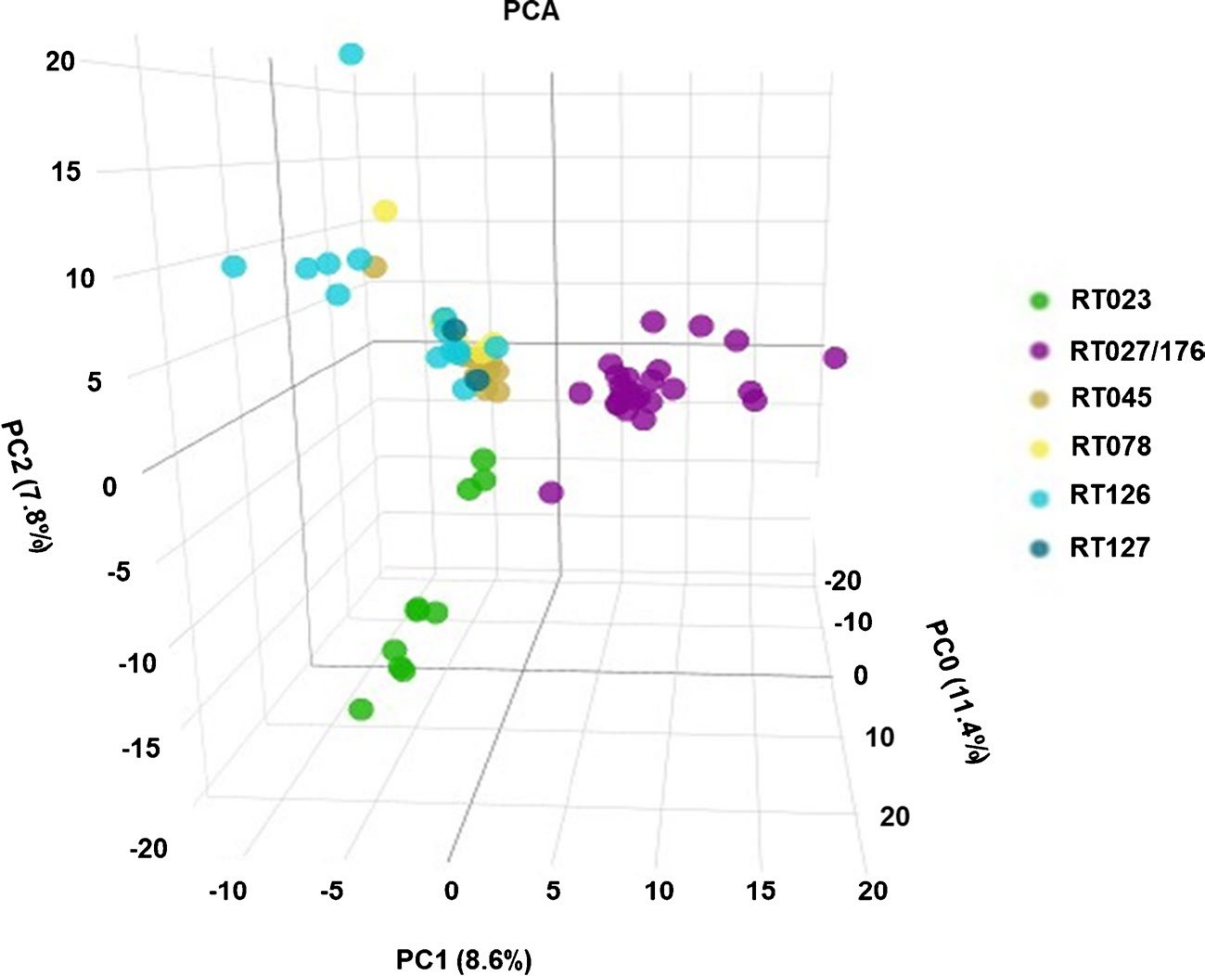
Classification among the HVR strains. Two-dimensional view of a principal component analysis (PCA) using total ion current normalization and peak detection with 1% threshold (TICp), separating RT027/176 (violet color) from RT045, RT078, RT126, RT127, and RT023

Given the promising separation of HVRTs and non-HVRTs by the RF and PLS-DA models, we wondered whether these two models could further discriminate between different HVRTs used in this study. However, when spectra of all isolates of the training set were included, no clear separation between specific HVRTs was attainable (Supplementary File S5). Thus, we next tested, if a better separation of certain HVRTs can be achieved by a two-step procedure, in which HVRTs were identified in a first step as described above. Next, we created a second peak matrix based on the average MALDI-TOF spectra of the training set HVRTs using the TICp method. With HVRTs’ peak matrix being used as input for PCA, three different clusters were observed (Fig. 4).

**Fig. 5:**
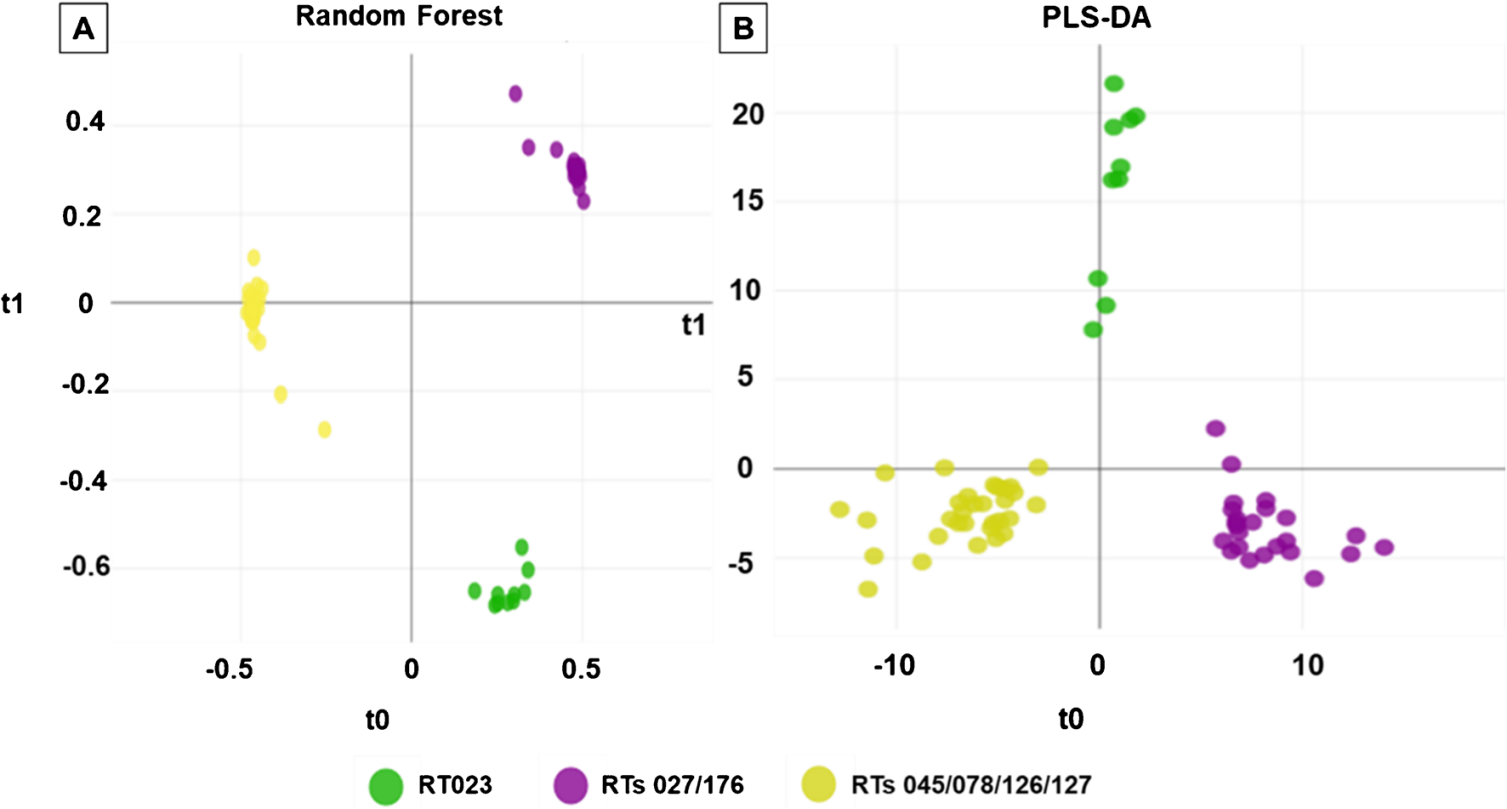
Classification of hypervirulent (HVR) *C. difficile* isolates using supervised ML algorithms. Random forest (RF) (**A**) and partial least squares–discriminant analysis (PLS-DA) (**B**). Each circle represents one *C. difficile* isolate. Isolates belonging to RT023 are indicated in green, while isolates of RT027/176 are depicted in violet. Other HVR RTs isolates are shown in yellow. RTs, ribotypes

One cluster encompassed RT023 isolates, another cluster comprised RT027/176 isolates, while isolates of RT045, RT078, RT126, and RT127 grouped together in a third cluster. RF and PLS-DA algorithms confirmed the initial PCA findings (Fig. 5).

10-fold cross-validation resulted in 100% accuracy for both models (Table 4 and Supplementary File S6).

**Table 4:** Classification of HVR RTs, confusion matrix of 10-fold cross-validation results: scores (in %) obtained with two (2) supervised ML algorithms (RF and PLS-DA). *HVR*, hypervirulent; *RT*, ribotypes

10-fold cross-validation (65 HVR isolates)				
Random forest (RF) and partial least square discriminant analysis (PLS-DA)				
Actual/predicted	RT023	RT027/176	RT045/078/126/127	% Correct
RT023	10	0	0	100%
RT027/176	0	24	0	100%
RT045/078/126/127	0	0	31	100%
				100% (accuracy)

External validation of the two prediction models was next performed using average spectra of all 39 HVRT isolates from the validation set (Table 1). Overall accuracies of 92.3% (RF) and 97.4% (PLS-DA) were achieved (Table 5). However, three RT023 isolates were misclassified as RT045/078/126/127 (RF), while only one RT078 isolate was misclassified as RT023 (PLS-DA) (Table 5 and Supplementary File S7).

**Table 5:** Classification of HVR RTs, confusion matrix of external validation results: scores (in %) obtained with two (2) supervised ML algorithms (RF and PLS-DA). *HVR*, hypervirulent; *RT*, ribotypes

External validation (39 isolates)				
Actual/predicted	RT023	RT027/176	RT045/078/126/127	% Correct
Random forest (RF)				
RT023	6	0	3	66.7%
RT027/176	0	7	0	100%
RT045/078/126/127	0	0	23	100%
				92.3% (accuracy)
Partial least square discriminant analysis (PLS-DA)				
RT023	9	0	0	100%
RT027/176	0	7	0	100%
RT045/078/126/127	1	0	22	95.7%
				97.4% (accuracy)

## Discussion

MALDI-TOF is a widely distributed, easy-to-use method for identifying bacterial species [11]. Timely subtyping of *C. difficile* is crucial for outbreak confirmation. Ribotyping and WGS [9, 10] are currently used for subtyping with higher costs compared to MALDI-TOF (~1.5$ and >200$ vs. 0,5$) [23–25].

However, with limitations, subtyping by MALDI-TOF is also possible. In particular, RT027/176 are one of the best-known RTs, which can be differentiated based on their protein extract-based MALDI-TOF spectra from other genotypes [17]. Other differentiable RTs include RT001 [14, 15], RT017 [16], and the HVRTs 078/126 [15]. It is unclear yet whether MALDI-TOF can be used to discriminate between HVRTs and non-HVRTs. Thus, the study’s aim was to test whether this might be achieved blended with ML.

We showed that protein extract-based MALDI-TOF spectra coupled with ML can indeed be used to distinguish between HVRTs and non-HVRTs circulating in Europe (accuracy >95%). Furthermore, subtyping of certain HVRTs (e.g., RT027/176 or RT023) was possible (100% accuracy, PLS-DA model), when a two-step procedure was applied. First, HVRTs were discriminated from non-HVRTs with a peak matrix containing isolates of both HVRTs and non-HVRTs and subsequently mapped against a second peak matrix consisting of HVRT isolates only. Nevertheless, this two-step procedure failed to separate certain HVRT isolates (RT045/078/126/127) from each other. Congruent with previous findings, RT027 and RT176 were indistinguishable [17]. RT023 identification might be of interest, as it is considered an emerging clade 3 strain [5].

MALDI-TOF HVRT identification represents a noteworthy option for rapid, preliminary surveillance and outbreak investigation as published for Italy and Brazil [14, 26]. It might estimate the potential transmission between patients, since some HVRTs are more likely to cause outbreaks [4]. However, any MALDI-TOF-based HVRT identification should be confirmed by other methods like WGS to allow a more accurate discrimination between clonal strains [27].

The study’s limitations are that subtyping of HVRTs was performed with 65 isolates as a training set, and for most of the HVRTs tested here, the number of isolates was comparably low (i.e., ≤10). To substantiate our hypothesis that MALDI-TOF/ML can be used to identify major HVRTs in Europe, it will be important to test additional isolates expanding the HVRT repertoire. Particularly, rarer HVRTs could be included, as they might be identifiable by MALDI-TOF/ML.

## Conclusion

MALDI-TOF/ML allowed to distinguish between HVRTs and non-HVRTs circulating in Europe with an accuracy >95% and can be used to separate certain HVRTs subgroups from each other (RT023, RT027/176, and RT045/078/126/127). Our findings suggest that this approach might offer a fast, reliable, and accessible tool for preliminary identification of major HVRTs circulating in Europe.

### Supplementary information

ESM 1 (DOCX 24 kb)

ESM 2 (XLSX 20 kb)

ESM 3 (DOCX 282 kb)

ESM 4 (DOCX 104 kb)

ESM 5 (DOCX 350 kb)

ESM 6 (DOCX 65 kb)

ESM 7 (DOCX 110 kb)

## Data Availability

Data are available on reasonable request from the corresponding author.
