# Supplementary material for: Discrimination between hypervirulent and non-hypervirulent ribotypes of Clostridioides difficile by MALDI-TOF mass spectrometry and machine learning
Source: Eur J Clin Microbiol Infect Dis. 2023 Sep 18;42(11):1373–81. doi: 10.1007/s10096-023-04665-y (PMC10587247; doi:10.1007/s10096-023-04665-y)
Supplement: Supplementary file 1 — (DOCX 24 kb) [file 10096_2023_4665_MOESM1_ESM.docx]

**Supplementary Table1:** 157 clinical strains of epidemiologically important ribotypes provided via the NRC *C. difficile* strain collection for the training phase:

Hypervirulent (HVR) ribotypes n=65

Non-Hypervirulent (Non-HVR) ribotypes n=92

| Ribotype | Number of isolates | Virulence | Strain Code |
| --- | --- | --- | --- |
| RT001* | 8 | Non-HVR | P3/ H2/ H13/ K1/ K42/ P26/ P42/ R34 |
| RT002* | 6 | Non-HVR | W41/ W65/ C59/ H16/ P2/ W28 |
| RT009 | 6 | Non-HVR | A69/ E73/ F68/ G41/ K26/ U45 |
| RT010* | 6 | Non-HVR | O59/O62/ O73/ O9/ P55/ P12 |
| RT012 | 6 | Non-HVR | D16/ X1-54/ X1-75/ X9-57/ X9-60/ CD630 |
| RT014* | 5 | Non-HVR | E20/ J59/ CN70/ V11/ L60 |
| RT017 | 7 | Non-HVR | B46/ M38/ R30/ X6-77/ X7-25/ L56/ S31 |
| RT018* | 9 | Non-HVR | G41/ H30/ U30/ X1-55/ X2-14/ X2-47/ X4-16/ X4-56/ Q57 |
| RT020* | 4 | Non-HVR | C23/ J58/ P1/ O10 |
| RT023^+^ | 10 | HVR | N22/ N23/ P17/ R18/ T32/ U60/ U67/ V80/ W36/W8 |
| RT027* | 14 | HVR | X5-51/ P47/ K27/ P72/ P75/ Q17/ Q41/ Q43/ Q50/ R20291/ S16/ W18/ Q69/ X5-41 |
| RT35 | 3 | Non-HVR | B63/ H66/ M37/ |
| RT045 | 10 | HVR | B25/ B65/ H56/ M9/ N63/ O52/ O56/ P63/ P71/ P73 |
| RT073 | 4 | Non-HVR | A10/A33/ D21/ F28 |
| RT078* | 5 | HVR | N71/ H35/ P79/ W62/ S79 |
| RT084 | 2 | Non-HVR | H46/ M47 |
| RT085 | 2 | Non-HVR | Q22/T23 |
| RT106 | 6 | Non-HVR | X5-29/ X5-55/ I73/ P45/ S35/ T69 |
| RT126 | 14 | HVR | F32/ M19/ M50/ M53/ N51/ N53/ N8/ O64/ Q63/ R4/ V62/ V66/ X5-30/ X6-74 |
| RT127 | 2 | HVR | G49/ U01 |
| RT140* | 11 | Non-HVR | D48/ E27/ P21/ P55/ R66/ S25/ S57/ T15/ T71/ B47/ V75 |
| RT176* | 10 | HVR | A1/ A3 / A5 / A7 / A9 / E6 / F31 / G62 / K44 / W74 |
| RT207 | 7 | Non-HVR | C48/ V31/ C74/ T79/ V10/ V27/ V30 |

*For these RTs according to Davies et al. 2016^[[1]](#footnote-1)^ European prevalence rates can be given. These are as follows: RT001 (11%); RT002 (4%), RT010 (3%) RT014/020 (10%), RT018 (3%), RT027 (19%), RT176 (2%).

^+^For RT023 in a study from UK, a prevalence of 2.4% was evident.^[[2]](#footnote-2)^

**Supplementary Table 2:** 83 clinical strains of various ribotypes provided via the NRC *C. difficile* strain collection for the validation phase:

Hypervirulent (HVR) ribotypes n=39

Non-Hypervirulent (Non-HVR) ribotypes n=44

| Ribotype | Number of isolates | Virulence | Sample IDs |
| --- | --- | --- | --- |
| RT001 | 5 | Non-HVR | C32/ E33/ F54/ G28/ L18 |
| RT002 | 7 | Non-HVR | E30/ M55/ P19/ P25/ X56/ XX1.71/ XX6.47 |
| RT009 | 1 | Non-HVR | XX2.25 |
| RT010 | 2 | Non-HVR | P8/ XX2.67 |
| RT014 | 5 | Non-HVR | C33/ E31/ F52/ H15/ H44 |
| RT017 | 6 | Non-HVR | J51/ J52/ J53/ J54/ J55/ J56 |
| RT018 | 3 | Non-HVR | N32/ XX3.81/ XX5.13 |
| RT020 | 9 | Non-HVR | F74/ G50/ H43/ H61/ J57/ L29/ M2/ M56/ XX2.24 |
| RT023 | 9 | HVR | H75/ J17/ X47/ XX59/ XX2.16/ XX2.34/ XX2.35/ XX4.78/ XX5.36 |
| RT027 | 7 | HVR | B12/ B40/ B64/ C6/ C12/ D4/ X51 |
| RT045 | 4 | HVR | C3/ J27/ Q5/ XX4.18 |
| RT078 | 16 | HVR | B74/ F10/ F78/ I7/ I37/ J38/ J59/ P9/ Q15/ Q66/ Q73/ XX3.5/ XX2.10/ XX2.23/ XX3.40/ XX4.45 |
| RT106 | 3 | Non-HVR | E38/ G64/ N39 |
| RT126 | 3 | HVR | C13/ I6/ XX1.68 |
| RT140 | 3 | Non-HVR | XX1.5/ XX1.53/ C27 |

1. Davies Kerrie A, Ashwin Helen, Longshaw Christopher M, Burns David A, Davis Georgina L, Wilcox Mark H, on behalf of the EUCLID study group. Diversity of *Clostridium difficile* PCR ribotypes in Europe: results from the European, multicentre, prospective, biannual, point-prevalence study of *Clostridium difficile* infection in hospitalised patients with diarrhoea (EUCLID), 2012 and 2013. Euro Surveill. 2016;21(29):pii=30294. https://doi.org/10.2807/1560-7917.ES.2016.21.29.30294 [↑](#footnote-ref-1)
2. Shaw HA, Preston MD, Vendrik KEW, Cairns MD, Browne HP, Stabler RA, Crobach MJT, Corver J, Pituch H, Ingebretsen A, Pirmohamed M, Faulds-Pain A, Valiente E, Lawley TD, Fairweather NF, Kuijper EJ, Wren BW. The recent emergence of a highly related virulent *Clostridium difficile* clade with unique characteristics. Clin Microbiol Infect. 2020 Apr;26(4):492-498. doi: 10.1016/j.cmi.2019.09.004. Epub 2019 Sep 13. PMID: 31525517; PMCID: PMC7167513. [↑](#footnote-ref-2)
