# Supplementary material for: Discrimination between hypervirulent and non-hypervirulent ribotypes of Clostridioides difficile by MALDI-TOF mass spectrometry and machine learning
Source: Eur J Clin Microbiol Infect Dis. 2023 Sep 18;42(11):1373–81. doi: 10.1007/s10096-023-04665-y (PMC10587247; doi:10.1007/s10096-023-04665-y)
Supplement: Supplementary file 3 — (DOCX 282 kb) [file 10096_2023_4665_MOESM3_ESM.docx]

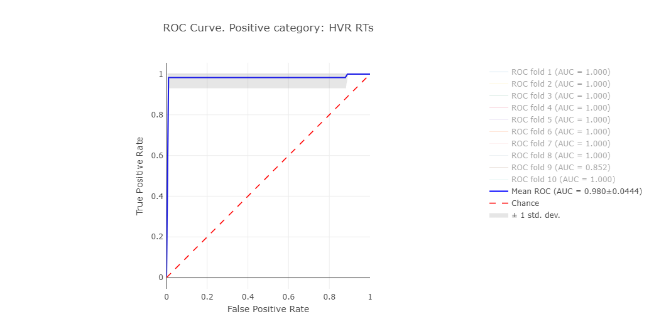


**A)**


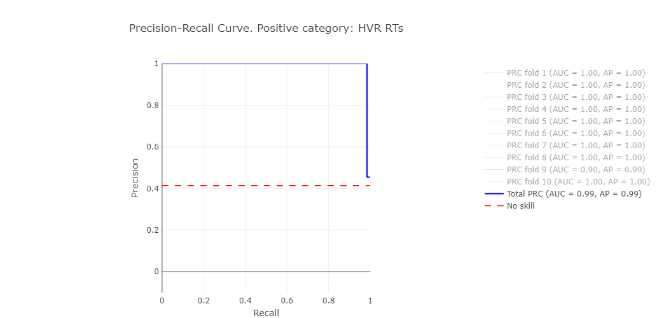


**B)**

Classification of HVR vs Non-HVR: Performance of RF prediction model during 10-fold cross-validation. (A) ROC and (B) PR curves.


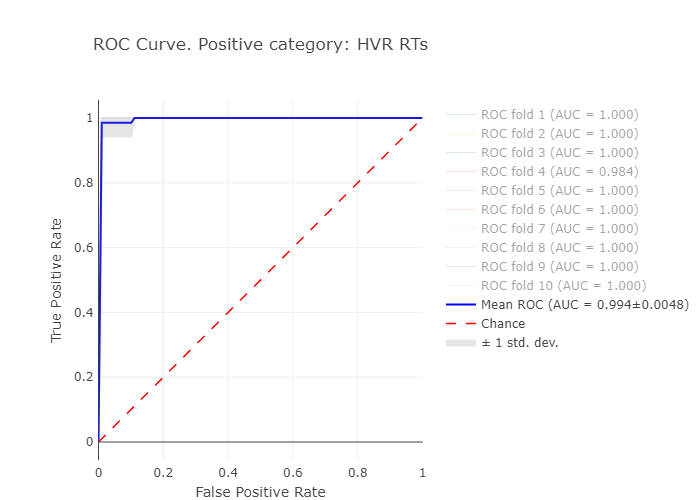

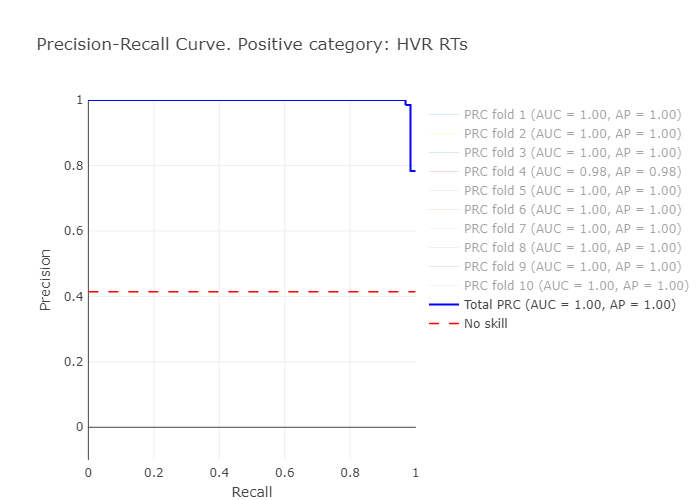


**B)**

**A)**

Classification of HVR vs Non-HVR: Performance of PLS-DA prediction model during 10-fold cross-validation. (A) ROC and (B) PR curves.


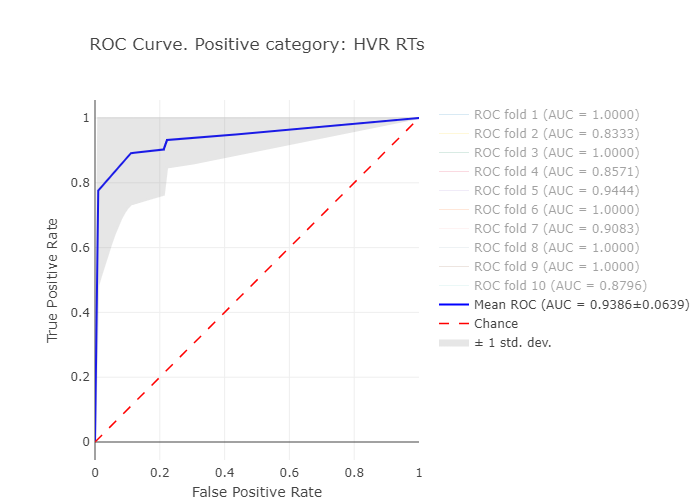

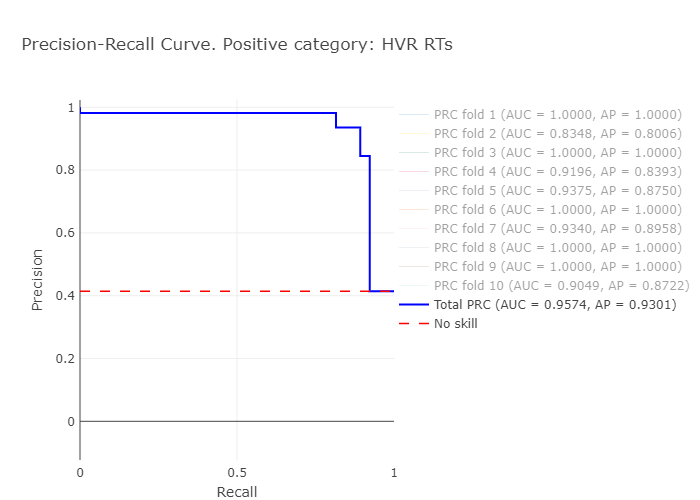


**B)**

**A)**

Classification of HVR vs Non-HVR: Performance of KNN prediction model during 10-fold cross-validation. (A) ROC and (B) PR curves.


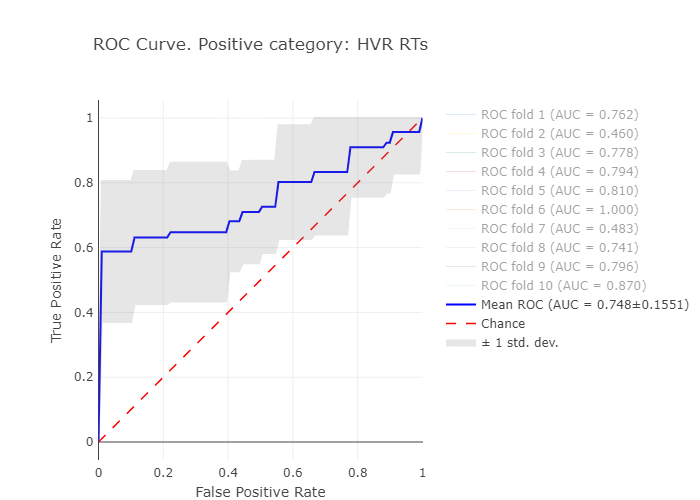

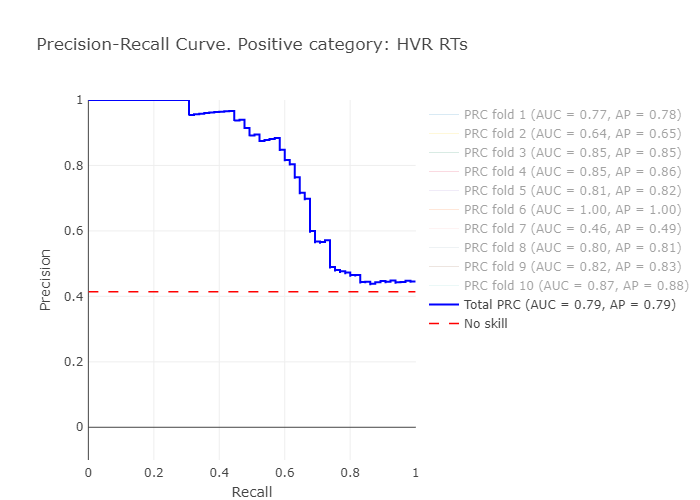


**B)**

**A)**

Classification of HVR vs Non-HVR: Performance of SVM prediction model during 10-fold cross-validation. (A) ROC and (B) PR curves.
