# Supplementary material for: Discrimination between hypervirulent and non-hypervirulent ribotypes of Clostridioides difficile by MALDI-TOF mass spectrometry and machine learning
Source: Eur J Clin Microbiol Infect Dis. 2023 Sep 18;42(11):1373–81. doi: 10.1007/s10096-023-04665-y (PMC10587247; doi:10.1007/s10096-023-04665-y)

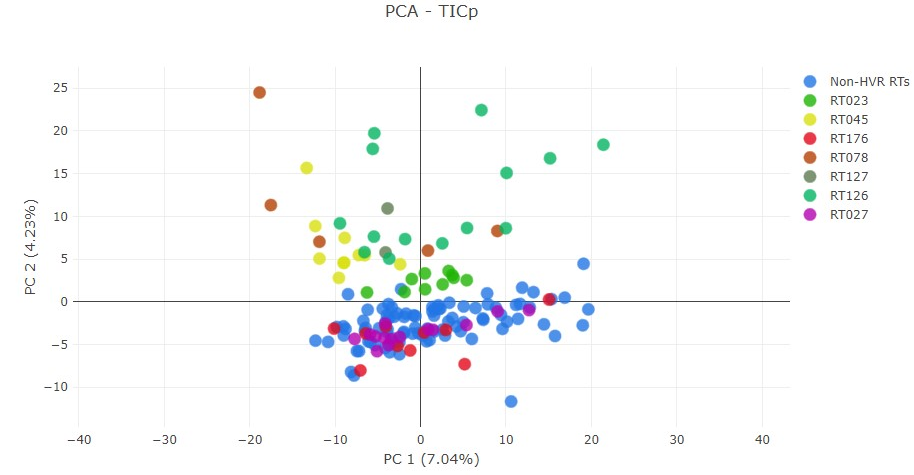
Classification applying an unsupervised algorithm: Two-dimensional view of a principal component analysis (PCA) using “threshold method” with normalization performed before peak finding (TICp). Each circle represented an individual *C. difficile* strain visualized with different colors associated with the RT group. Non-HVR RTs, non-hypervirulent ribotypes depicted in blue.


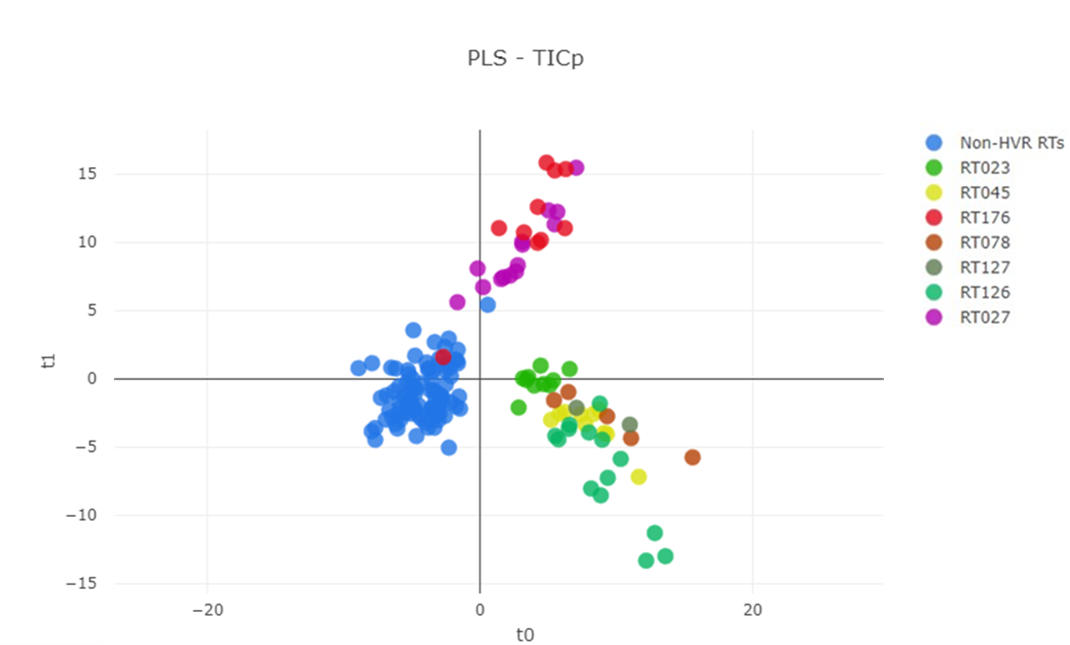


Classification applying a supervised algorithm: Two-dimensional view of a partial least square discriminant analysis (PLS-DA) using “threshold method” with normalization performed before peak finding (TICp). Each circle represented an individual *C. difficile* strain visualized with different colors associated with the RT group. Non-HVR RTs, non-hypervirulent ribotypes depicted in blue.

**PLS-DA: 10-fold cross validation**


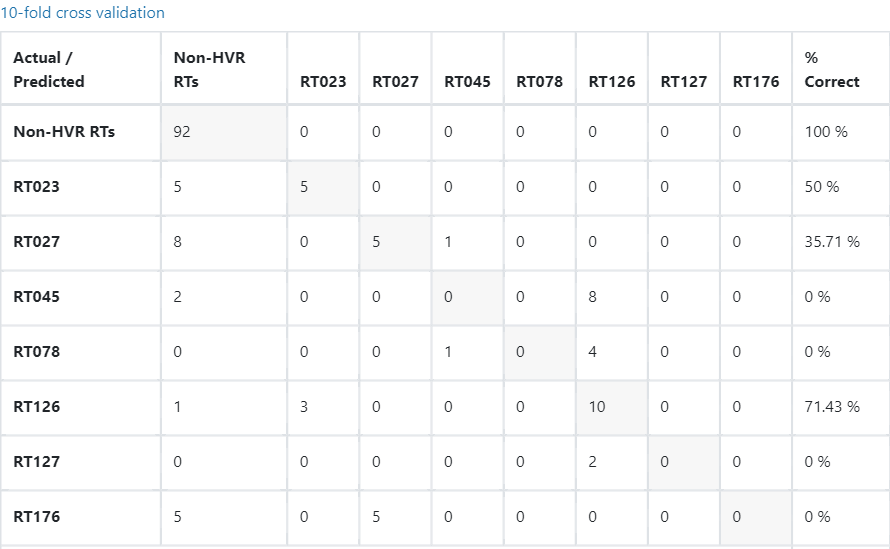

Supplement: Supplementary file 5 — (DOCX 350 kb) [file 10096_2023_4665_MOESM5_ESM.docx]
