# Supplementary material for: Discrimination between hypervirulent and non-hypervirulent ribotypes of Clostridioides difficile by MALDI-TOF mass spectrometry and machine learning
Source: Eur J Clin Microbiol Infect Dis. 2023 Sep 18;42(11):1373–81. doi: 10.1007/s10096-023-04665-y (PMC10587247; doi:10.1007/s10096-023-04665-y)
Supplement: Supplementary file 6 — (DOCX 65 kb) [file 10096_2023_4665_MOESM6_ESM.docx]

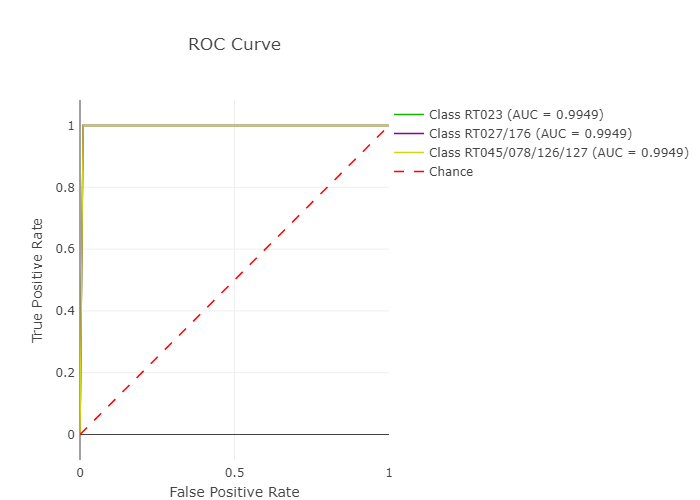


**A)**


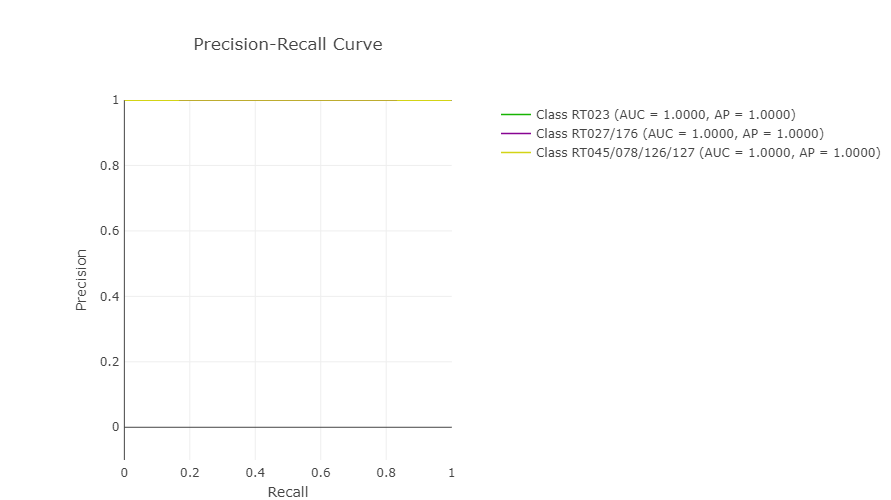


**B)**

Classification of HVR RTs: Performance of both RF and PLS-DA prediction models during 10-fold cross-validation. (A) ROC and (B) PR curves.
